# Persistent COVID-19-associated neurocognitive symptoms in non-hospitalized patients

**DOI:** 10.1007/s13365-021-00954-4

**Published:** 2021-02-02

**Authors:** Joanna Hellmuth, T. Allen Barnett, Breton M. Asken, J. Daniel Kelly, Leonel Torres, Melanie L. Stephens, Bryan Greenhouse, Jeffrey N. Martin, Felicia C. Chow, Steven G. Deeks, Meredith Greene, Bruce L. Miller, Wesley Annan, Timothy J. Henrich, Michael J. Peluso

**Affiliations:** 1 Memory and Aging Center, Department of Neurology, University of California, San Francisco, CA USA; 2 Weill Institute for Neuroscience, Department of Neurology, University of California, San Francisco, USA; 3 School of Medicine, University of California, San Francisco, CA USA; 4 Department of Epidemiology & Biostatistics, University of California, San Francisco, CA USA; 5 Division of Experimental Medicine, Department of Medicine, University of California, San Francisco, CA USA; 6 Division of Infectious Diseases, Department of Medicine, University of California, San Francisco, CA USA; 7 Division of HIV, Infectious Diseases, and Global Medicine, Department of Medicine, University of California, San Francisco, CA USA; 8 Division of Geriatrics, Department of Medicine, University of California, San Francisco, CA USA

**Keywords:** COVID-19, Cognitive changes, Brain fog, Outpatient COVID-19

## Abstract

As cases of coronavirus disease 2019 (COVID-19) mount worldwide, attention is needed on potential long-term neurologic impacts for the majority of patients who experience mild to moderate illness managed as outpatients. To date, there has not been discussion of persistent neurocognitive deficits in patients with milder COVID-19. We present two cases of non-hospitalized patients recovering from COVID-19 with persistent neurocognitive symptoms. Commonly used cognitive screens were normal, while more detailed testing revealed working memory and executive functioning deficits. An observational cohort study of individuals recovering from COVID-19 (14 or more days following symptom onset) identified that among the first 100 individuals enrolled, 14 were non-hospitalized patients reporting persistent cognitive issues. These 14 participants had a median age of 39 years (interquartile range: 35–56), and cognitive symptoms were present for at least a median of 98 days (interquartile range: 71–120 following acute COVID-19 symptoms); no participants with follow-up evaluation reported symptom resolution. We discuss potential mechanisms to be explored in future studies, including direct viral effects, indirect consequences of immune activation, and immune dysregulation causing auto-antibody production.

## Introduction

Viral infections can cause cognitive symptoms through direct viral effects on the central nervous system (CNS) or through indirect phenomena. For SARS-CoV-2 infection, current descriptions of cognitive issues focus on hospitalized patients with severe disease (Helms et al. 2020; Varatharaj et al. 2020). These cases may be confounded by prolonged hospitalization and severe systemic illness and do not necessarily suggest direct or indirect viral effects on the CNS. Additionally, these patients do not represent the majority of people infected with SARS-CoV-2 who develop mild to moderate symptoms managed in outpatient settings. We describe two patients with persistent neurocognitive symptoms following SARS-CoV-2 infection who were not hospitalized during acute illness. Both display deficits on neuropsychological testing that do not correlate with mood, sleep, or fatigue issues. We also present early findings on reported cognitive symptoms in an observational cohort of SARS-CoV-2 infected patients in recovery.

## Case 1

A 33-year-old otherwise healthy, high-functioning Hispanic woman was evaluated in our memory clinic. She initially developed neck pain, fatigue, fever, cough, myalgias, and non-migrainous headaches. Within days she lost her sense of smell and taste and a SARS-CoV-2 PCR test returned positive. Cognitive symptoms started during the first week of symptoms and include difficulty focusing, forgetfulness that improves with cues, and problems processing and keeping track of information. Two weeks later, she developed left arm parasthesias and neuropathic pain. Her sense of smell and taste slowly improved. She returned to work 114 days after symptom onset and noted workplace challenges due to cognitive issues, requiring adaptive strategies.

She has no major medical issues other than attention-deficit hyperactivity disorder (ADHD, inattentive type) diagnosed 12 years prior by neuropsychological testing, and took no scheduled psychoactive medications. She holds a master’s degree, and there were no issues with substance use. She denied any depressive symptoms impairing functional abilities. A comprehensive neurologic evaluation 149 days after symptom onset revealed improving left whole arm parasthesias and dysesthesias and 4+/5 strength in the left 5th digit abduction. She scored 30/30 on the Montreal Cognitive Assessment (MoCA). Detailed neuropsychological testing revealed mild disorganization and inefficient, error-prone task execution (Table 1). Compared with testing 12 years prior, she displayed deficits in working memory (3-min recall of a complex figure was 6th percentile; previously 25th percentile) and digit span backwards (4, low average; previously 6, average) with high average attentional skills (Guilmette et al. 2020). Other cognitive domains appeared normal. Laboratory testing revealed normal values for vitamin B12, TSH, RPR, SPEP, lactate dehydrogenase, D-dimer, and interleukin-6; HIV testing was negative. Cerebrospinal fluid revealed no white blood cells, and normal glucose, protein (18 mg/dL), CSF/serum albumin index (2.3), IgG index (0.5), and oligoclonal bands (a single band in CSF and none in serum). A brain and cervical spine MRI with contrast were unremarkable.

**Table 1:** Case 1, neuropsychological testing performance 149 days after first COVID-19 symptoms

Domain	Test	Raw score	Performance descriptor
Global cognition	Montreal Cognitive Assessment	30/30	-
Memory	California Verbal Learning Test-3 (16-word)		
	Immediate recall trials 1–5, total	48/80	Average
	Short delay free recall	10/16	Average
	Long delay free recall	13/16	Average
	Rey-Osterreith Complex Figure		
	3-min delay	16/36	Below average^a^
	30-min delay	21.5/36	Average
Attention/working memory	WAIS-IV Digit Span		
	Forward span (longest)	8	High average
	Backward span (longest)	4	Low average^b^
Fluency/rapid generativity	D-KEFS Letter Fluency		
	F, A, S words	40 (0)	Average
	D-KEFS Category Fluency		
	Animals + boys’ names	42 (0)	Average
	Fruits/furniture switching	18 (0)	Above average
	D-KEFS Design Fluency		
	Filled dots	12 (0)	High average
	Empty dots	11 (1)	Average
	Switching dots	6 (7)	Average
Processing speed/set-shifting	WAIS-IV Coding	69 (0)	Average
	D-KEFS Trail Making		
	Number sequencing	22″ (0)	High average
	Letter sequencing	28″ (0)	Average
	Number-letter switching	64″ (1)	Average
	D-KEFS Color-Word Interference		
	Color naming	28″ (0)	Average
	Word naming	24″ (1)	Average
	Inhibition	44″ (4)	High average
	Inhibition/switching	77″ (2)	Low average
Visuospatial	NAB Visual Discrimination	17/18	High average
	Rey-Osterreith Complex Figure Copy	33/36	Low average

For D-KEFS fluency and WAIS-IV coding tasks, raw scores represent total correct responses. For D-KEFS Trail Making and Color-Word Interference, raw scores reflect time in seconds needed to complete the task. Numbers in parentheses reflect errors made on each test. D-KEFS tests with multiple conditions are organized by progressive complexity with increasing cognitive demand. Raw scores were converted to demographically adjusted standardized scores, and then a performance descriptor based on percentile. We followed demographic corrections based on the data provided within each specific test manual. Descriptor terms reflect American Academy of Clinical Neuropsychology consensus descriptors for standardized score ranges: Above average: 91st–97th percentiles. High average: 75th–90th percentiles. Average: 25th–74th percentiles. Low average: 9th–24th percentiles. Below average: 2nd–8th percentiles (Guilmette et al. 2020). The neuropsychological testing performed 12 years prior did not include many of the raw scores and had few directly comparable tests, limiting full presentation of these data.

*WAIS* Wechsler Adult Intelligence Scale, *D-KEFS* Delis-Kaplan Executive Function System, *NAB* Neuropsychological Assessment Battery.

^a^Neuropsychological testing 12 years prior did include 3-min recall of a complex figure, on which she scored 25th percentile (average), compared with 6th percentile (below average) on this testing.

^b^Testing 12 years prior on digit span backwards was 6 (average, no percentile provided) compared with the 16th percentile (low average) on this testing

## Case 2

A 56-year-old otherwise healthy, high-functioning White woman was evaluated by telemedicine 37 days after symptom onset for PCR-confirmed COVID-19. She initially developed loss of smell and taste, fatigue, diarrhea, and dyspnea; these symptoms later improved. During acute COVID-19 she experienced 2 days of a neurogenic bladder, persisting symptoms of a lower limb radiculopathy or neuropathy, and difficulty focusing. With return to work she noticed word finding difficulties, inefficient learning, and decreased organization leading to missed deadlines. She scored 30/30 on the Mini-Mental State Exam (MMSE). Digit span forward was 7 (average; measure of attention), and digit span backwards was 3 (below expectations; measure of working memory). Letter and category fluency were normal. Neurocognitive symptoms have persisted at least 72 days after symptom onset; further work up is pending. She was not prescribed any medications, there were no issues with substance use, and she holds a bachelor’s degree. She denied any depressive symptoms impairing functional abilities.

## Observational Study Data

The Long-term Impact of Infection with Novel Coronavirus (LIINC) study in San Francisco, CA, prospectively enrolls adults in recovery from COVID-19 at least 14 days from symptom onset with a documented positive SARS-CoV-2 nucleic acid amplification test (“Long-term Impact of Infection with Novel Coronavirus” 2020). Participants are evaluated at study entry, at approximately 1 month, and again 120 days after symptom onset. Cognitive symptoms were ascertained in the first 40 participants by asking about any new or worsening of pre-existing symptoms; subsequent participants were specifically asked about new or worsening concentration, thinking, or memory issues. In the first 100 participants, 20 reported cognitive issues during one or more study visit. Twelve participants reported cognitive symptoms that were present at the initial visit and only one (8.3%) reported symptom resolution at a follow-up. Eight participants did not report cognitive symptoms at the initial visit but did at a follow up. These 20 participants reporting cognitive symptoms had a median age of 41 years (interquartile range: 36–55), 50% (10) were female, with 65% (13) identifying as White, 30% (6) identifying as Latina/a ethnicity, 10% (2) identifying as Black, and 5% (1) identifying as American Indian. Forty percent (8) reported no medical comorbidities. Eighty percent (16) were employed, which is similar to the 74% employment among the first 100 participants. Fourteen of these 20 were never hospitalized for COVID-19, and in half (8) cognitive symptoms started during acute COVID-19. Eleven of these 14 have had follow-up visits, during which all reported persistent cognitive symptoms. Among these 14 non-hospitalized participants, cognitive symptoms were present for at least a median 98 days (interquartile range: 71–120; range: 48–142) following COVID-19 symptom onset. These 14 never hospitalized participants had a median age of 39 years (interquartile range 35–56), and their demographics did not differ significantly from the hospitalized participants.

## Discussion

In addition to reported symptoms, we identified measurable executive functioning deficits in young and middle-aged adults who were never hospitalized during acute COVID-19. The observational study data suggests cognitive symptoms may be common in patients recovering from COVID-19 and last months or longer after acute illness. Interestingly, these cognitive deficits were not captured by the MMSE or MoCA, common cognitive screens, suggesting neuropsychological testing is warranted (Folstein et al. 1975; Nasreddine et al. 2005). Future work would benefit from systematic cognitive assessments of ambulatory COVID-19 patients.

Several hypothesized mechanisms may contribute to COVID-19-associated neurocognitive symptoms. Not all COVID-19 patients with severe neurologic sequelae have evidence of SARS-CoV-2 in cerebrospinal fluid, suggesting both direct viral and indirect mechanisms contribute to CNS dysfunction (Espíndola et al. 2020; Ellul et al. 2020). The observed executive functioning deficits are clinically similar to HIV-associated neurocognitive disorder, which has known associations with increased inflammation and immune activation (Yuan et al. 2015; Cysique et al. 2006; Heaton et al. 2011). SARS-CoV-2 infection also has associated patterns of elevated immune activation, although relationships with cognitive functioning are not yet known (Lucas et al. 2020; Laing et al. 2020; Del Valle et al. 2020). Immune dysregulation leading to anti-neuronal antibody production could cause cognitive deficits, although the first patient’s normal IgG index and oligoclonal bands argue against intrathecal antibody production that would be expected with a CNS immune response to SARS-CoV-2. It is not known whether patients with COVID-19-associated neurocognitive symptoms display evidence of CNS damage. Elevated plasma biomarkers of astrocytic and neuronal injury were not found in one report of non-hospitalized COVID-19 patients; however, these cases were not selected for the presence of cognitive symptoms (Kanberg et al. 2020). Noting the first patient’s ADHD diagnosis, fronto-executive network differences may form a selective vulnerability increasing the risk for cognitive symptoms.

We observed COVID-19-associated neurocognitive symptoms in young and middle aged adults who were not hospitalized. Further work is needed to establish symptom prevalence, affected populations, spectrum of clinical involvement, natural history, and underlying mechanisms.
